# Ruptured Pulmonary Hydatid Cyst Complicated by COVID-19

**DOI:** 10.4269/ajtmh.22-0173

**Published:** 2022-05-31

**Authors:** Recep Tekin, Serdar Onat, Rojbin Ceylan Tekin

**Affiliations:** ^1^ Department of Infectious Diseases and Clinical Microbiology, Faculty of Medicine, Dicle University, Diyarbakir, Turkey;; ^2^ Department of Thoracic Surgery, Faculty of Medicine, Dicle University, Diyarbakir, Turkey;; ^3^ Department of Radiology, Mardin State Hospital, Mardin, Turkey

**Figure 1.**
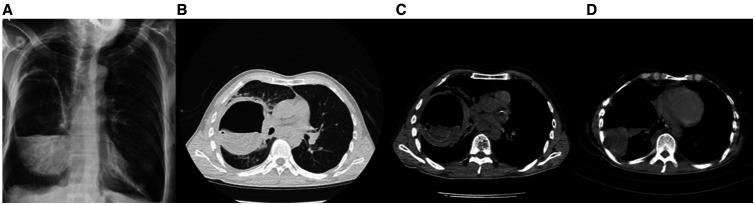
(**A**) Radiological image showed a cavitary lesion, which is considered a ruptured hydatid cyst. (**B**–**D**) Noncontrast chest computed tomography scan showed an air-containing cystic lesion with an internal undulating and collapsed germinative membranes representing detached membranes of hydatid cyst.

**Figure 2.**
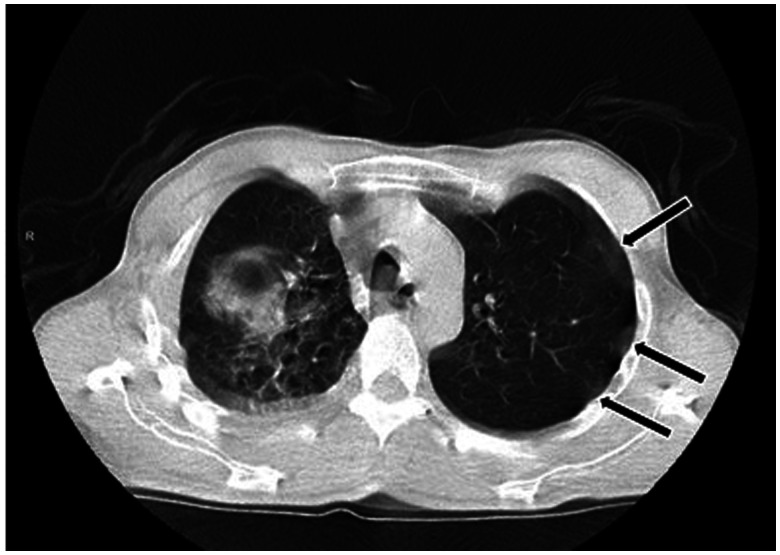
Axial reformatted unenhanced thorax computed tomography image demonstrates subpleural ground-glass opacities (arrows) in the bilateral lung.

**Figure 3.**
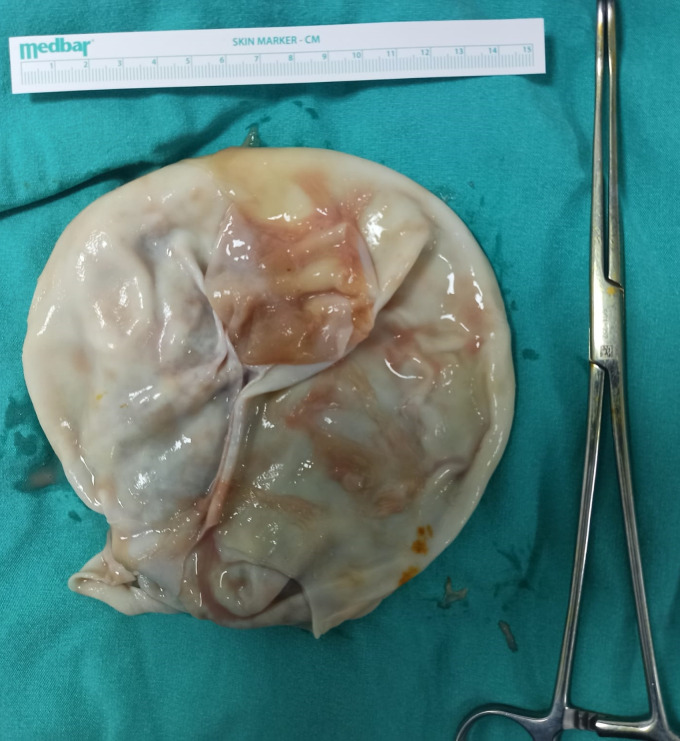
Gross examination showed removed cyst. This figure appears in color at www.ajtmh.org.

A 51-year-old man with a 1-day history of dyspnea; cough; and production of yellowish, foul-smelling, and salty sputum was admitted to the emergence department. Chest computed tomography (CT) scan revealed a voluminous cavitary lesion in the middle part of the right lung containing irregular, serpiginous intracavitary material compatible with the free-floating membrane of a hydatid cyst (Figure 1). The patient lived in a region endemic for hydatid disease. Eosinophilia in a peripheral blood sample and serum IgG against *Echinococcus granulosus* was positive at 1/5,120 titer on an immunofluorescence assay test. On the basis of the clinical, laboratory, and radiological findings, ruptured pulmonary hydatid cyst was diagnosed. On the second day of hospitalization, he reported fever, myalgia, and sore throat. A polymerase chain reaction test for SARS-CoV-2 ribonucleic acid from the nasopharyngeal swab confirmed the diagnosis of COVID-19. Chest CT scan was highly suggestive of COVID-19. Axial and coronal reformatted unenhanced thorax CT image demonstrates subpleural ground-glass opacities and vascular dilatation (arrows) in the bilateral lung (Figure 2). The patient underwent enucleation of the hydatid cysts with capitonnage as one-stage posterolateral thoracotomy (Figure 3). Histopathological examination of cysts confirmed the diagnosis of hydatid disease. It is rare for an individual patient to have both ruptured pulmonary hydatid cyst and COVID-19.^1^^,^^2^ COVID-19 during ruptured pulmonary hydatid cyst is potentially serious for the patient. Surgery combined with medical therapy remains the standard form of treatment.^3^ High clinical suspicion and early imaging diagnosis of this condition can enable clinicians to pursue more aggressive treatment options to reduce fatal outcomes.^4^^,^^5^ Hydatid cyst should be kept in mind in the differential diagnosis of the patients presenting with COVID-19, particularly in those who live in endemic areas.
